# Genomic predictors of testosterone levels are associated with muscle fiber size and strength

**DOI:** 10.1007/s00421-021-04851-w

**Published:** 2021-11-18

**Authors:** João Paulo L. F. Guilherme, Ekaterina A. Semenova, Oleg V. Borisov, Andrey K. Larin, Ethan Moreland, Edward V. Generozov, Ildus I. Ahmetov

**Affiliations:** 1 Laboratory of Applied Nutrition and Metabolism, School of Physical Education and Sport, University of São Paulo, São Paulo, Brazil; 2 Department of Molecular Biology and Genetics, Federal Research and Clinical Center of Physical-Chemical Medicine of Federal Medical Biological Agency, Moscow, Russia; 3 Research Institute of Physical Culture and Sport, Volga Region State University of Physical Culture, Sport and Tourism, Kazan, Russia; 4 Research Institute for Sport and Exercise Sciences, Liverpool John Moores University, Liverpool, UK; 5 Department of Physical Education, Plekhanov Russian University of Economics, Moscow, Russia; 6 Laboratory of Molecular Genetics, Kazan State Medical University, Kazan, Russia

**Keywords:** Elite athletes, Polymorphism, Genetics, Hormones, Skeletal muscle, Athletic performance

## Abstract

**Purpose:**

Circulating testosterone levels are a heritable trait with anabolic properties in various tissues, including skeletal muscle. So far, hundreds of single nucleotide polymorphisms (SNPs) associated with testosterone levels have been identified in nonathletic populations. The aim of the present study was to test the association of 822 testosterone-increasing SNPs with muscle-related traits (muscle fiber size, fat-free mass and handgrip strength) and to validate the identified SNPs in independent cohorts of strength and power athletes.

**Methods:**

One hundred and forty-eight physically active individuals (47 females, 101 males) were assessed for cross-sectional area (CSA) of fast-twitch muscle fibers. Significant SNPs were further assessed for fat-free mass and handgrip strength in > 354,000 participants from the UK Biobank cohort. The validation cohorts included Russian elite athletes.

**Results:**

From an initial panel of 822 SNPs, we identified five testosterone-increasing alleles (*DOCK3* rs77031559 G, *ESR1* rs190930099 G, *GLIS3* rs34706136 TG, *GRAMD1B* rs850294 T, *TRAIP* rs62260729 C) nominally associated (*P* < 0.05) with CSA of fast-twitch muscle fibers, fat-free mass and handgrip strength. Based on these five SNPs, the number of testosterone-increasing alleles was positively associated with testosterone levels in male athletes (*P* = 0.048) and greater strength performance in weightlifters (*P* = 0.017). Moreover, the proportion of participants with ≥ 2 testosterone-increasing alleles was higher in power athletes compared to controls (68.9 vs. 55.6%; *P* = 0.012).

**Conclusion:**

Testosterone-related SNPs are associated with muscle fiber size, fat-free mass and strength, which combined can partially contribute to a greater predisposition to strength/power sports.

**Supplementary Information:**

The online version contains supplementary material available at 10.1007/s00421-021-04851-w.

## Introduction

Testosterone is an anabolic–androgenic steroid hormone produced mainly in Leydig cells of the testes in men and the ovary and the adrenal cortex in women. Testosterone plays an integral role in the development and maintenance of male characteristics, including the development of primary and secondary sex characteristics and the maintenance of the reproductive system. In addition, testosterone plays a clear role on several non-reproductive tissues, regardless of gender. In skeletal muscle, testosterone and its metabolite, dihydrotestosterone, have a well-defined anabolic property, mainly through an increase in protein synthesis via the activation of the mammalian target of rapamycin (mTOR) pathway together with the androgen receptor (AR) signaling (Basualto-Alarcon et al. 2013; Zeng et al. 2017). Other anabolic or anti-catabolic mechanisms have also been proposed (Dubois et al. 2012), all suggestive that testosterone plays an important role in muscle mass regulation.

Testosterone administration has been shown to increase muscle mass and strength in a dose-dependent manner in young and older men (Bhasin et al. 2001; Bhasin et al. 2005) and in young women (Horwath et al. 2020). Given that muscle hypertrophy (and the increase in muscle function it brings) has a performance-enhancing effect in sports that depend on strength and power, higher levels of testosterone create an advantage (Wood and Stanton 2012). Not surprisingly, testosterone is the most common form of doping in sport; however, it should be mentioned that due to the dynamic regulation of its endogenous production, testosterone concentrations may vary considerably within and among individuals. There is a strong heritability for serum testosterone, with genetic factors accounting for 40–70% of the variation in testosterone levels in men (Travison et al. 2014) and 65% in women (Hong et al. 2001).

It is possible that individuals who have higher levels of endogenous testosterone are more predisposed to certain power sports. In an assessment of a large cohort of elite male athletes, sprinters showed higher free testosterone levels than athletes in other sports (e.g., long-distance runners) (Bermon and Garnier 2017). Similarly, in an assessment of a large cohort of elite female athletes, sprinters showed higher testosterone levels than long-distance runners (Bermon et al. 2014). Higher testosterone levels in female sprinters can contribute to athletic success, allowing them to reach a higher competitive level (Ahmetov et al. 2020). Indeed, female athletes with higher free testosterone performed better in 400 to 800-m sprinting events, hammer throw and pole vault compared with female competitors with lower free testosterone (Bermon and Garnier 2017). Testosterone is a contributory trait in the complex nature of athletic phenotypes, and can influence athletic performance (e.g., increased neuronal activity, bone growth and hemoglobin levels) (Wood and Stanton 2012).

A recent genome-wide association study (GWAS) provided a number of single nucleotide polymorphisms (SNPs) associated with higher total and bioavailable testosterone levels in men and women (Ruth et al. 2020). These innate characteristics may lead to interindividual differences in hormone levels capable of influencing testosterone-related phenotypes, such as predisposition to increase muscle mass and strength. However, with the exception of a polymorphism in the *AR* gene (with the potential to affect testosterone levels) (Guilherme et al. 2021b), the interaction of testosterone-increasing alleles with muscle mass and function remains to be investigated.

Testosterone-induced gains in muscle size were associated with a significant increase in muscle fiber cross-sectional area (CSA). In young healthy, eugonadal men treated with graded doses of testosterone, the increases in muscle volume are associated with concentration-dependent increases in CSA of both type I and type II muscle fibers (Sinha-Hikim et al. 2002). In young healthy, physically active women (20–35 years) treated with testosterone cream for 10 weeks, muscle hypertrophy was primarily driven by increases in CSA of type II fibers (Horwath et al. 2020). Overall, the CSA of muscle fibers correlates positively with strength variables, especially when it comes to type II (fast-twitch) fibers. Since fast-twitch fibers are required in high-energy movement tasks such as sprinting or weightlifting, their CSA is of vital importance for power athletes.

Muscle CSA can be affected by numerous environmental factors, but it is also highly determined by genetic factors. One can inherit genetic polymorphisms which make muscle hypertrophy easier than others who do not possess those polymorphisms. Some individual SNPs were associated with larger CSA of fast-twitch muscle fibers (Ahmetov et al. 2008; Broos et al. 2016; Grishina et al. 2019; Seaborne et al. 2019). These SNPs, associated with a larger CSA of fast-twitch fibers, were also more frequent in strength and power athletes, which suggest a favourable genetic profile. Athletic phenotypes (including muscle strength and power) are polygenic in nature, which implies that multiple polymorphisms influence these athletic phenotype (Guilherme and Lancha 2020; Moreland et al. 2020).

Although there is a relationship between testosterone levels and muscle mass regulation, the shared genetic background between testosterone-increasing alleles and muscle CSA is poorly understood. Therefore, the purpose of the present study was to explore whether GWAS-identified testosterone-increasing SNPs (Ruth et al. 2020) were associated with muscle fiber size (CSA of fast-twitch muscle fibers), fat-free mass and muscle strength. To validate the SNPs that met the selection criteria (testosterone-increasing alleles associated with muscle-related traits), independent athlete cohorts were assessed using a polygenic approach. The combined association of the selected SNPs (based on the number of favorable alleles) was assessed for testosterone levels in male athletes, strength performance in elite weightlifters and prevalence in strength and power athletes.

## Materials and methods

### Participants and ethical approval

The analysis of the CSA of fast-twitch muscle fibers was carried out in 148 physically active participants with mixed training (i.e., aerobic + resistance) background (Table 1). They were classified according to their training frequency as mildly active (2 training sessions per week), moderately active (3–4 training sessions per week), highly active (5–7 training sessions per week) or extremely active (two training sessions per day). Testosterone-increasing SNPs associated with the CSA of fast-twitch muscle fibers were subsequently tested for associations with fat-free mass and handgrip strength in the UK Biobank—a prospective population-based study of > 354,000 individuals (summary statistics is available from https://genetics.opentargets.org/).

**Table 1:** Characteristics of physically active participants who underwent muscle biopsy to determine the CSA of fast-twitch muscle fibers

Group	*N*	Age	Height (cm)	Weight (kg)	Training age (years)^†^	CSA of fast-twitch fibers (μm^2^)
Females	47	28.0 ± 1.1	167.2 ± 0.8	59.7 ± 0.8	10.7 ± 1.0	4,305 ± 202
Males	101	31.1 ± 0.8	180.2 ± 0.6	80.3 ± 1.0	11.7 ± 0.9	5,925 ± 164

^**†**^Training age was expressed as years of regular training

Following this two-stage association analysis, the selected SNPs (using a polygenic approach) were tested in three independent athlete cohorts: (i) 49 male athletes with power component in their athletic performance (age 22.8 ± 0.7 years; 1 100-m runner, 4 badminton players, 7 baseball players, 4 boxers, 5 canoeists, 8 figure skaters, 2 kayakers, 6 rowers, 3 speed skaters, 4 volleyball players and 5 wrestlers) were tested for testosterone levels; (ii) 50 weightlifters (29 males, age 23.7 ± 0.7 years; 21 females, age 22.5 ± 0.8 years) were tested for strength performance in official competitions; (iii) 222 international-level power athletes (126 males, age 23.6 ± 0.4 years; 96 females, age 22.8 ± 0.5 years) were tested for the prevalence of favorable alleles compared to 151 non-athlete controls (120 males and 31 females, age 44.7 ± 4.0 years). The power athlete group was composed of 67 strength athletes (17 powerlifters and 50 weightlifters), 82 speed-strength athletes (15 alpine skiers, 3 arm-wrestlers, 29 climbers, 4 heptathletes and decathletes, 22 jumpers and 9 throwers) and 73 sprinters (25 100- to 400-m runners, 19 200- to 500-m kayakers and 29 500- to 1000-m speed skaters). Athletes were Russian national team members who had never tested positive for doping. Controls were healthy unrelated Russians without any competitive sport experience.

**Fig. 1:**
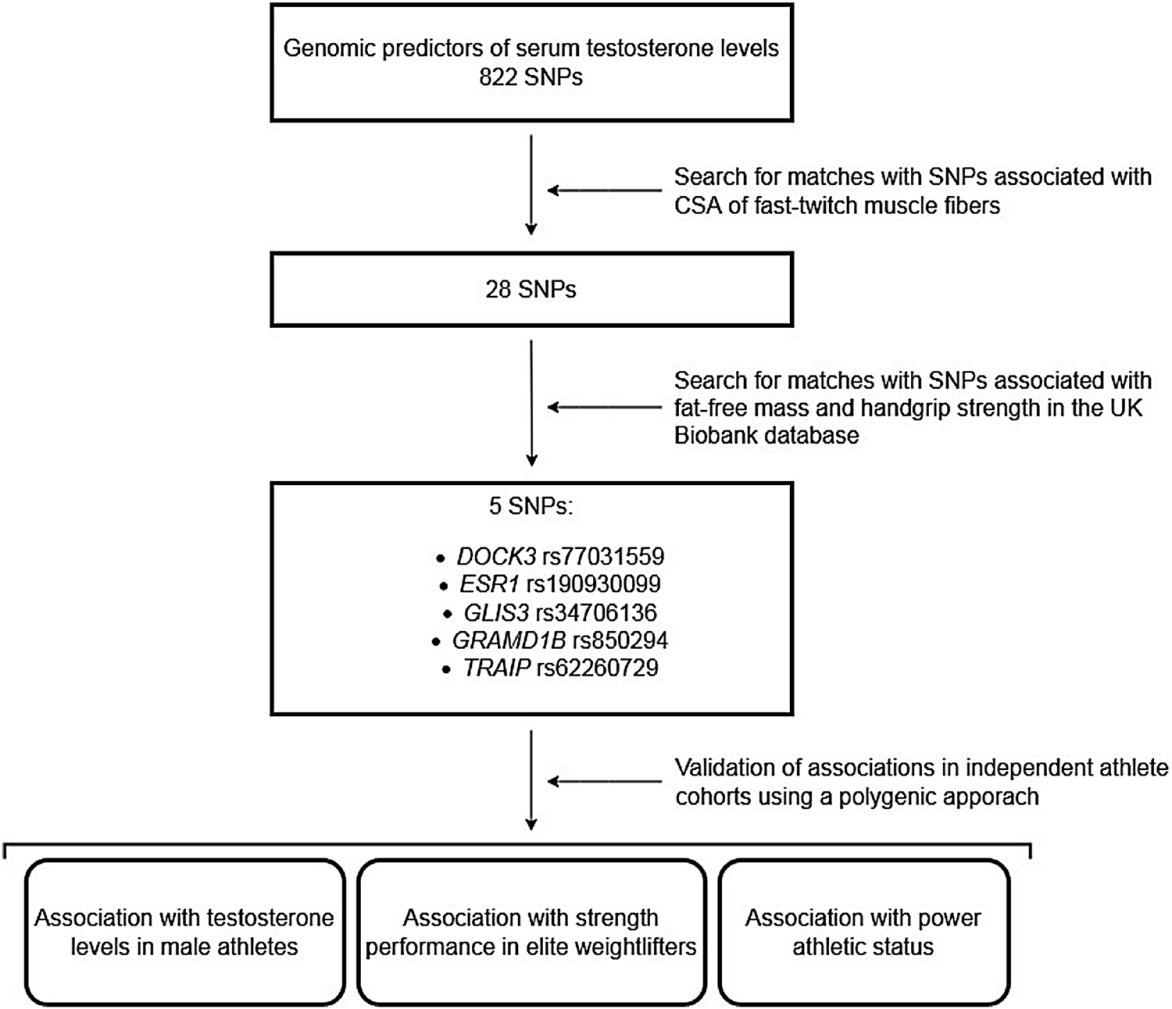
Study design showing the selection process for significant SNPs and testosterone-increasing alleles

A flow diagram displaying the selection process of the significant SNPs is shown in Fig. 1. This study was conducted in accordance with the Declaration of Helsinki and was approved by the Ethics Committee of the Federal Research and Clinical Center of Physical–chemical Medicine. Written informed consent was obtained from each participant.

### Muscle biopsy and determination of the CSA of fast-twitch muscle fibers

Vastus lateralis samples of 148 physically active participants were obtained from the left leg using the modified Bergström needle procedure with aspiration under local anaesthesia with 2% lidocaine solution. Prior to analysis, samples were frozen in liquid nitrogen and stored at − 80 °C. Serial cross-sections (7 μm) were obtained from frozen samples using an ultratom (Leica Microsystems, Germany). Sections were thaw-mounted on Polysine glass slides, maintained at room temperature (RT) for 15 min and incubated in PBS (3 × 5 min). The sections were then incubated at RT in primary antibodies against slow or fast isoforms of the myosin heavy chains (M8421, 1:5000; M4276; 1:600, respectively; Sigma-Aldrich, USA) for 1 h and incubated in PBS (3 × 5 min). Next, the sections were incubated at RT in secondary antibodies conjugated with FITC (F0257; 1:100; Sigma-Aldrich) for 1 h. The antibodies were removed, and the sections washed in PBS (3 × 5 min), placed in mounting media and covered with a cover slip. Images were captured by fluorescent microscope (Eclipse Ti-U, Nikon, Japan). All analyzed images contained 329 ± 14 fibers. The ratio of the number of stained fibers to the total fiber number was calculated. Fibers stained in serial sections with antibodies against slow and fast isoforms were considered hybrid fibers.

### Hormone levels and strength performance assessment

Resting testosterone levels were examined in serum of athletes. A total of 10 mL of venous blood were collected the morning after an overnight fast and sleep in tubes containing EDTA and placed at 4 °C until processing (blood was collected at least 15 h after the last training). Testosterone was analyzed on a microplate spectrophotometer (Bio-Rad, Hercules, CA, USA) using an enzyme immunoassay test (Alkor-Bio, St Petersburg, Russia).

Evaluation of strength in weightlifters was computed based on their performance in the snatch, and clean and jerk (best results in official competitions, including Olympic Games, European and World Championships). The total weight lifted (in kg) is multiplied by the Wilks Coefficient (Coeff) to find the standard amount lifted normalized across all body weights, as previously described (Grishina et al. 2019).

### DNA extraction and genotyping

Molecular genetic analysis was performed with DNA samples obtained from leukocytes (venous blood). DNA extraction and purification were performed using a commercial kit according to the manufacturer’s instructions (Technoclon, Moscow, Russia). Genotyping of SNPs was majorly performed using microarray technology, as previously described (Guilherme et al. 2021a).

### Statistical analyses

Statistical analyses were conducted using PLINK 1.9, R (3.4.3), and GraphPad InStat (GraphPad Software, Inc., USA). Haplotype phasing before imputation was performed using SHAPEIT. Imputation was performed using IMPUTE2. For phasing and imputation, we used 1000 Genomes Phase 3 data as a reference panel and imputed the variants with a frequency higher than 0.1% in the reference panel. Variants imputed with low certainty (info score < 0.6) were filtered out after imputation. Pearson’s correlation assessed the relationships between the number of favorable alleles (polygenic analysis) and different phenotypes. Allele frequencies between athletes and controls were compared using χ^2^ tests. All calculations were performed adjusting for covariates (muscle fiber size: principal component analysis (PCA), age, sex, physical activity and training type; handgrip strength: PCA, age, sex; fat-free mass: PCA, age, sex; testosterone levels in men: age; strength performance in weightlifters: age, sex). In particular, the search for association between 822 SNPs and CSA of fast-twitch muscle fibers was performed using logistic regression analysis adjusted for covariates. Data of testosterone levels were used from the study involving 425,097 UK Biobank participants by Ruth et al. (2020). Data of handgrip strength were used from the study involving 359,729 UK Biobank participants. Data of fat-free mass were used from the study involving 354,808 UK Biobank participants. Summary statistics for handgrip strength and fat-free mass are available from https://genetics.opentargets.org/. *P* values < 0.05 were considered statistically significant.

## Results

In the first stage, we tested the hypothesis that genome-wide significant testosterone-increasing alleles are associated with muscle fiber size. For this, we selected a panel of 855 SNPs (Supplementary Table 1) from the study by Ruth et al. (Ruth et al. 2020). However, the set of testosterone-increasing alleles included 822 SNPs, because 33 SNPs had directionally opposing effects between sexes and were not included in the present study. Of the 822 SNPs, 31 were nominally associated (*P* < 0.05) with CSA of fast-twitch muscle fibers (adjusted for covariates), with the same direction of association (i.e., testosterone-increasing alleles were associated with increased CSA). After exclusion of SNPs localized in the X chromosome (only autosomal chromosomes were evaluated), the set of testosterone-increasing alleles associated with muscle fiber size included 28 SNPs (Supplementary Table 2).

Next, using UK Biobank database, we have identified that of the 28 SNPs, 5 testosterone-increasing alleles (*DOCK3* rs77031559 G, *ESR1* rs190930099 G, *GLIS3* rs34706136 TG, *GRAMD1B* rs850294 T, *TRAIP* rs62260729 C) were also nominally associated (*P* < 0.05) with increased handgrip strength and fat-free mass in 359,729 and 354,808 individuals, respectively (Table 2).

**Table 2:** SNPs associated with testosterone levels, CSA of fast-twitch muscle fibers, handgrip strength and fat-free mass in different cohorts

Gene	SNP	Effect allele	Testosterone		CSA of FT muscle fibers		Handgrip strength		Fat-free mass	
			Beta	*P*	Beta	*P*	Beta	*P*	Beta	*P*
*ESR1*	rs190930099	G	0.135	8.1 × 10^–11^	2123.1	0.028	0.367	0.0024	0.485	0.0000013
*GLIS3*	rs34706136	TG	0.012	2.7 × 10^–8^	361.1	0.044	0.09	0.0000015	0.02	0.011
*TRAIP*	rs62260729	C	0.014	3.0 × 10^–10^	407.8	0.033	0.048	0.011	0.054	0.00067
*DOCK3*	rs77031559	G	0.027	9.2 × 10^–9^	939.5	0.016	0.072	0.045	0.101	0.0007
*GRAMD1B*	rs850294	T	0.034	5.2 × 10^–18^	824.8	0.011	0.055	0.0499	0.025	0.038

Effect allele: allele associated with increased values of testosterone levels, CSA of fast-twitch muscle fibers, handgrip strength, and fat-free mass

Although only two associations (rs34706136 for handgrip strength and rs190930099 for fat-free mass) passed Bonferroni correction for multiple testing (i.e. *P* value = 0.05/822 SNPs * 3 traits (CSA of muscle fibers, handgrip strength, fat-free mass) = 0.00002), we felt justified to use all 5 SNPs in the polygenic analysis given that we used SNPs already discovered independently via GWAS of testosterone levels at genome-wide significance (Ruth et al. 2020). To validate the five SNPs associated with testosterone levels and muscle-related traits, we performed a series of studies in independent cohorts of athletes using a polygenic approach (i.e., the combined association of the 5 SNPs), as shown in Fig. 1. We classified all participants according to the number of testosterone-increasing alleles they possessed, that is, carriers of *DOCK3* rs77031559 AA, *ESR1* rs190930099 AA, *GLIS3* rs34706136 TT, *GRAMD1B* rs850294 CC, and *TRAIP* rs62260729 TT genotypes had zero testosterone-increasing alleles, whereas participants with *DOCK3* rs77031559 GG, *ESR1* rs190930099 GG, *GLIS3* rs34706136 TG/TG, *GRAMD1B* rs850294 TT, and *TRAIP* rs62260729 CC genotypes had 10 testosterone-increasing alleles (heterozygous genotypes were computed with intermediate scores).

The number of testosterone-increasing alleles was positively associated with testosterone levels in elite male athletes (*r* = 0.28; *P* = 0.048) and with greater strength performance (total lifts in snatch and clean and jerk adjusted for sex and weight) in elite weightlifters (*r* = 0.34; *P* = 0.017). None of the participants (athletes or controls) had the maximum number of testosterone-increasing alleles (range from 0 to 6 alleles). However, the proportion of participants with a high number of testosterone-increasing alleles (i.e., ≥ 2 alleles instead of 0–1 allele) was greater among power athletes compared to controls (68.9 vs 55.6%; odds ratio (OR) = 1.8, *P* = 0.012). Table 3 summarizes the findings of the polygenic analysis.

**Table 3:** Comparison between carriers of a low number (0–1) of testosterone-increasing alleles and carriers of a high number (≥ 2) of testosterone-increasing alleles (polygenic analysis) in athlete cohorts

Parameters	0–1 allele	≥ 2 alleles	*P* value
Testosterone levels, nmol/l^†^	22.1 (4.6)	23.4 (4.6)	0.048
Weightlifting performance, points^†^	235.3 (17.9)	245.7 (23.6)	0.017
Proportion of power athletes^‡^, %	31.1	68.9	0.012
Proportion of controls^‡^, %	44.4	55.6	**–**

^†^Data are Mean (SD)

^‡^The minor allele frequencies (MAFs, %) in the power athlete group were: 6.0 (*DOCK3* rs77031559 G), 1.6 (*ESR1* rs190930099 G), 45.0 (*GLIS3* rs34706136 TG), 10.7 (*GRAMD1B* rs850294 T) and 39.6 (*TRAIP* rs62260729 C), while the MAFs (%) in the control group were: 6.3 (*DOCK3* rs77031559 G), 0.0 (*ESR1* rs190930099 G), 43.0 (*GLIS3* rs34706136 TG), 9.3 (*GRAMD1B* rs850294 T) and 35.1 (*TRAIP* rs62260729 C)

## Discussion

To our knowledge, this is the first study aimed towards identifying the shared genetic background between testosterone-increasing alleles, muscle traits and athletic performance. We identified five SNPs that were associated with testosterone levels, CSA of fast-twitch muscle fibers, fat-free-mas and handgrip strength.

The five identified SNPs are located in introns of genes that have multiple functions in relation to the endocrine system, metabolism and cellular function. More specifically, *DOCK3* (Dedicator Of Cytokinesis 3) encodes a protein involved in the regulation of actin cytoskeleton and cell adhesion receptors; *ESR1* (Estrogen Receptor 1) encodes a nuclear receptor for estrogen that controls many cellular processes including growth and differentiation; *GLIS3* (GLIS Family Zinc Finger 3) encodes a protein involved in the development of pancreatic beta cells, the thyroid, liver and kidney; *GRAMD1B* (GRAM Domain Containing 1B) encodes a protein that plays a crucial role in cholesterol homeostasis; *TRAIP* (TRAF Interacting Protein) encodes a protein involved in cell activation and protection against apoptosis. Interestingly, in an assessment of young, healthy men who underwent 10 weeks of resistance training, three of these genes alter their expression in skeletal muscle in response to resistance training compared to pre-training (*ESR1* and *GLIS*) or endurance training (*GRAMD1B*) (Vissing and Schjerling 2014). Moreover, according to the GTEx portal (https://gtexportal.org), two SNPs (*TRAIP* rs62260729 and *DOCK3* rs77031559) are functional and influence the expression of several genes in various tissues, including testis, adrenal gland and skeletal muscle—all important in terms of testosterone production, physical performance and training responses. Noteworthy, *TRAIP* rs62260729 C allele is associated with increased expression of *CDHR4* gene, which has its expression increased in response to resistance exercise (Vissing and Schjerling 2014). More details of gene function, effects of SNPs and gene expression following resistance training are shown in Supplementary Table 3.

The physiological implication of higher testosterone levels in skeletal muscle is the maintenance or increase (hypertrophy) of skeletal muscle mass, and a subsequent indirect increase in muscle strength, which can be advantageous for power athletes. Larger fast-twitch fibers lead to a larger whole muscle and a greater muscle volume. Top-level sprinters, for example, have a higher fat-free mass due to greater muscle volume, which can explain almost half (47.5%) of the variability in sprint performance (Miller et al. 2020). A larger muscle volume is able to generate stronger and more powerful contractions, resulting in greater sprint speed (Miller et al. 2020). Although it is well established that muscle volume can be affected by numerous environmental factors, genetic variability between individuals likely determines the extension of muscle adaptation. In the present study, those with ≥ 2 testosterone-increasing alleles (also associated with muscle fiber size, fat-free mass and handgrip strength) had greater strength (weightlifting) performance in competition and are 1.8 times more likely to be an elite power athlete. These 5 SNPs can be part of a favorable polygenic profile for muscle hypertrophy and strength performance (the innate predisposition to complex phenotypes involves the sum of several common polymorphisms). However, the biological role of these genes and SNPs in skeletal muscle is not fully understood.

The *ESR1* gene is probably the one that has the most evidence in relation to skeletal muscle growth. Animal studies have shown that *ESR1* elimination resulted in an increase in *tibialis anterior* muscle mass (a fast-twitch muscle) (Brown et al. 2009). In turn, an assessment of the pre-training skeletal muscle transcriptome of healthy men and women clustered as non-responders (Non), modest responders (Mod), and extreme responders (Xtr) to resistance training (based on differential magnitudes of myofiber hypertrophy), there was a stepwise increase in *ESR1* expression from Non to Mod to Xtr, suggesting that estrogen signaling may be important for increased hypertrophic capacity (Thalacker-Mercer et al. 2013). Our findings support the relevance of the *ESR1* gene for muscle hypertrophy, with the rs190930099 G allele likely playing a role. We did not assess the participants' estrogen levels, but a recent study showed that a cluster of testosterone-increasing alleles also increased estradiol levels in men (consistent with the physiological conversion of testosterone to estrogen) (Ruth et al. 2020). Of note, the *ESR1* gene is expressed in skeletal muscle of men and women (Lemoine et al. 2003) and is therefore a tissue target for estrogen action.

Two other identified genes (*CDHR4* [near *TRAIP*] and *GLIS3*) are also responsive to resistance training (Vissing and Schjerling, 2014). The *CDHR4* gene [near *TRAIP*] was found to be necessary for axon guidance and cell migration in GABAergic neuromuscular junction development (Ackley 2014), which seems to play a signaling role in the contractile activity of skeletal muscle (Lenina et al. 2019). The *GLIS3* gene have been associated with a decreased risk of knee osteoarthritis (Zhang et al. 2021), but its relationship with muscle performance remains to be further investigated. Although there is no evidence of gene regulation by resistance training, the *DOCK3* may also be a contributing factor. The *DOCK3* gene promotes axonal outgrowth via cytoskeleton reorganization and plays an important role in the muscle tone (Helbig et al. 2017). Furthermore, the DOCK family of proteins has been shown to bind to regulators of PTEN/AKT signaling (Jungmichel et al. 2014), an important signaling for muscle hypertrophy. Molecular inhibition of *DOCK3* in skeletal muscle increases phosphorylated AKT levels, which influences the muscle morphology and function (Alexander et al. 2014). In general, the SNPs in the aforementioned genes were associated with muscle hypertrophy and strength, but still need to elucidate whether they are acting directly on skeletal muscle.

The only identified gene that has a known direct relationship to testosterone production was *GRAMD1B*, which belongs to a family of sterol-binding proteins. Steroidogenic cells take up cholesterol to initiate steroidogenesis (i.e., cholesterol is a substrate for testosterone biosynthesis), and the *GRAMD1B* gene assists in the transfer of cholesterol from the plasma membrane to the endoplasmic reticulum, where steroid hormones are produced (Larsen et al. 2020). GRAMD1 proteins facilitate the movement of accessible plasma membrane cholesterol to the endoplasmic reticulum, and cells that lack GRAMD1 proteins result in less efficient cholesterol transfer (Naito et al. 2019). It is unknown how the *GRAMD1B* rs850294 affects testosterone levels; however, this gene has been shown to be expressed differently between resistance and endurance training—opposite ends of the training-induced muscle adaptation continuum (Vissing and Schjerling 2014). It is well established that more intense exercises (common in a resistance training program) induce a greater increase in circulating testosterone levels (D'Andrea et al. 2020), as well as greater muscle hypertrophy (Lasevicius et al. 2018). In line with this, muscle power-related SNPs, such as *ACTN3* R577X, have previously been associated with higher testosterone levels (Ahmetov et al. 2014; Pimenta et al. 2012).

It has been shown that there is a positive change in lean mass per unit higher of testosterone (bioavailable testosterone in men and testosterone total in women) (Ruth et al. 2020). Testosterone, like all other hormones, act in an integrated communication network responsible for modulating cellular signaling. Therefore, the combination of the 5 SNPs identified in this study could somehow favor the hypertrophy of fast-twitch muscle fibers and strength performance. Here, we explore the influence of these SNPs (under a polygenic profile) in the context of sporting excellence, however, the issue is also relevant for clinical conditions affecting muscle mass.

Our study does have limitations. First, none of the associations between SNPs and CSA of muscle fibers passed correction for multiple testing, but we felt justified to use five SNPs in the polygenic analysis given that we used SNPs which were initially found in GWAS, meaning that in the discovery phase (Ruth et al. 2020) these SNPs have passed correction for multiple testing at genome-wide significance (*P* < 5.0·10^–8^). It is common not to adjust for multiple comparisons in the validation phase to prevent the loss of potentially important findings (Duncan et al. 2019; Wood et al. 2014). Second, there may be other SNPs acting on the traits of interest that the present study was unable to detect. It is worth mentioning that this study was an initial approach to align a large number of testosterone-related SNPs with physiological and functional data in elite athletes. Replication and functional studies with independent and larger samples will be beneficial to confirm the present findings. Another limitation of our study was that underlying mechanisms explaining the results have not been assessed. An observed statistical association between a genetic marker and a phenotype does not necessarily mean a causal relationship. Further mechanistic investigations are warranted to elucidate the possible mechanisms related to these markers.

In conclusion, the relationship between testosterone levels and muscle fiber size can partly be explained by shared genetic variants. We identified five testosterone-increasing alleles (*DOCK3* rs77031559 G, *ESR1* rs190930099 G, *GLIS3* rs34706136 TG, *GRAMD1B* rs850294 T, *TRAIP* rs62260729 C) that were also associated with CSA of fast-twitch muscle fibers, fat-free mass and handgrip strength. Based on these five SNPs, the number of testosterone-increasing alleles was positively associated with testosterone levels and weightlifting performance, as well as participants with ≥ 2 favorable alleles were overrepresented in power athletes. While many more genetic factors undoubtedly remain undiscovered (Ahmetov et al. 2021), these five provide a basis on which future, more comprehensive, genetic assessments might augment systems of identifying and nurturing talent in elite power sports.

## Supplementary Information

Below is the link to the electronic supplementary material.

Supplementary file1 (XLSX 52 KB)

## Abbreviations

AR: Androgen receptor
CSA: Cross-sectional area of muscle fibers
*DOCK3*: Dedicator of cytokinesis 3 gene
DNA: Deoxyribonucleic acid
DHT: Dihydrotestosterone
*ESR1*: Estrogen receptor 1 gene
GWAS: Genome-wide association study
*GLIS3*: GLIS family zinc finger 3 gene
*GRAMD1B*: GRAM domain-containing 1B gene
mTOR: Mammalian target of rapamycin
PCA: Principal component analysis
SNPs: Single-nucleotide polymorphisms
TRAIP: TRAF interacting protein
